# Interpersonal Mindfulness, Intergroup Anxiety, and Intercultural Communication Effectiveness Among International Students Studying in Russia

**DOI:** 10.3389/fpsyg.2022.841361

**Published:** 2022-05-13

**Authors:** Oleg Khukhlaev, Irina Novikova, Anna Chernaya

**Affiliations:** ^1^Department of Cross-Cultural Psychology and Multicultural Education, Moscow State University of Psychology and Education (MSUPE), Moscow, Russia; ^2^Department of Psychology and Pedagogics, Peoples’ Friendship University of Russia (RUDN University), Moscow, Russia; ^3^Developmental Psychology Chair, South Federal University, Rostov-on-Don, Russia

**Keywords:** interpersonal mindfulness, intergroup anxiety, intercultural effectiveness, Anxiety/Uncertainty Management theory, international students, *Interpersonal Mindfulness Scale*, Russian universities, intercultural communication

## Abstract

In modern psychology, mindfulness is an important resource for psychological well-being and intergroup relations, but its role in intercultural communication effectiveness has not been sufficiently studied. This research aims to identify the interrelationship between interpersonal mindfulness, intergroup anxiety, and intercultural communication effectiveness among international students. The sample includes 337 (*M*_age_ = 22.93, *SD* = 3.11) international students (41.5% of females) from different countries studying in Russian Universities. Interpersonal mindfulness was measured using the *Interpersonal Mindfulness Scale*, Intergroup anxiety using ten items adapted from Stephan and Stephan and used in Gudykunst and Nishida, and Intercultural communication effectiveness using the eight items adapted from Gudykunst’s *Perceived Effectiveness of Communication measure.* Descriptive analysis, correlations, and mediation analyses were used to process the data. The research findings showed that interpersonal mindfulness has both a direct effect on intercultural communication effectiveness and a mediation effect on intercultural communication effectiveness through intergroup anxiety among the international students.

## Introduction

Academic mobility of students remains one of the current trends in the development of education worldwide, despite rising international tensions and restrictive measures imposed in connection with the COVID-19 pandemic (Abramova et al., 2021; Gritsenko et al., 2021; Kanjera, 2021). While studying abroad, international students face several problems in intercultural adjustment and communication (Chebotareva, 2011; Novikova and Novikov, 2015; Bierwiaczonek and Waldzus, 2016; Khukhlaev et al., 2020; Aresi et al., 2021; Bui et al., 2021; Pekerti et al., 2021; Mao et al., 2022). Therefore, an important task of psychological science is the search for basic psychological mechanisms of the effectiveness of intercultural communication and using them to support international students.

There are numerous studies on the factors and predictors of the effectiveness of intercultural communication among international students in a new socio-cultural environment, including socio-demographic characteristics, health status, personality traits, tolerance, identity, metastereotypes, cognitive abilities, intercultural competence; cultural intelligence, and so on (Güngör and Bornstein, 2009; de Araujo, 2011; Neuliep, 2012; Jenny, 2013; Bücker et al., 2014; Bierwiaczonek and Waldzus, 2016; Presbitero and Attar, 2018; Thomas, 2018; Solhaug and Kristensen, 2020; Gritsenko et al., 2021; Ma and Xia, 2021; Matera and Catania, 2021; Cao and Meng, 2022; Xiong et al., 2022). The listed variables have a different impact on intercultural communication depending on external and internal conditions, which in turn sets the task of finding new predictors of the effectiveness of intercultural communication among international students. As intercultural communication is a special case of interpersonal communication, it is advisable to consider the already studied factors to improve the interpersonal communication to check their effectiveness in the intercultural communication.

One of the novel and recently proven resources to increase the effectiveness of interpersonal communication is interpersonal mindfulness. Burgoon et al. (2000) believed that being mindful while interacting with others likely fosters effective communication, which is critical for the healthy functioning of interpersonal relationships. Pratscher et al. (2019) later proposed the concept of interpersonal mindfulness: “when people are interpersonally mindful, they maintain a receptive awareness of what is going on during interpersonal interactions, moment-by-moment. They are aware of their own thoughts, emotions, feelings, bodily sensations, experiences, and intentions as the interaction occurs. At the same time, they pay attention to what seems to be going on with the other person, picking up clues through, not only what is said or done but also the other person’s apparent mood, verbal tone, and body language” (Pratscher et al., 2019, p. 1445). Because interpersonal mindfulness occurs in the moment-to-moment unfolding of social interactions, it is strongly related to communication outcomes (Pratscher et al., 2019). In this regard, it is logical to assume that mindfulness in interpersonal relationships with a representative of a different culture also increases the effectiveness of communication. However, no research has explored the impact of the interpersonal mindfulness to intercultural communication.

Based on these facts, this study primarily aimed to examine whether interpersonal mindfulness was related to intercultural communication effectiveness, and to explain this mechanism both theoretically and empirically.

### Interpersonal Mindfulness in Intercultural Communication

As mentioned above, there have been no studies on the contribution of interpersonal mindfulness to the effectiveness of intercultural communication; however, interpersonal mindfulness can be considered the mindfulness manifestation in interpersonal relationships.

The association of mindfulness with the effectiveness of interpersonal communication has been proven in studies (Burgoon et al., 2000; Lillis and Hayes, 2007; Karremans et al., 2017; Don, 2020), and the crucial role of mindfulness in interaction with people from a different culture has also been shown. Therefore, Gudykunst (1995, 1998) in his Anxiety/Uncertainty Management (AUM) theory incorporated the concepts of mindfulness and intercultural communication effectiveness; suggesting that communication is ineffective if people are not mindful during intercultural communication (Gudykunst and Nishida, 2001). According to the central tenets of AUM, the more an individual can regulate and manage uncertainty and anxiety, the more mindfulness they can achieve, leading to communication effectiveness (Neuliep, 2012).

Further, Thomas (2006) considered the concept of mindfulness as a key component that links knowledge with behavioral capability. Mindfulness as a metacognitive strategy focuses attention on the knowledge of culture and the processes of cultural influence as well as on an individual’s motives, goals, emotions, and external stimuli. Mindfulness is the central point in the Ting-Toomey and Dorjee (2015) intercultural and intergroup communication competence model that promotes increased culture-sensitive and identity-sensitive knowledge, open-hearted attitudes, and communication skill sets.

Previous empirical studies have supported the idea of positive impact of mindfulness on intercultural interaction. Systematic review of the mindfulness-intergroup literature found that mindfulness has the potential to assuage intergroup bias toward a variety of social groups (e.g., racial, gender, religious, and socioeconomic) (Oyler et al., 2021).

However, despite relatively well-researched role of mindfulness in intergroup relations, its influence on intercultural communication effectiveness has not been studied in depth. Jankowski and Sandage (2014) discovered the association between meditative prayer and intercultural competence. Mindfulness may play an important role in intercultural adjustment of international students; higher mindfulness being associated with better sociocultural adjustment during cross-cultural transitions (Kashima et al., 2017). Nadeem and Koschmann (2021) found that when Pakistani students were more mindful, they could better manage their levels of anxiety and uncertainty; having a positive impact on their intercultural communication effectiveness.

Thus, interpersonal mindfulness can be considered as a significant predictor of the effectiveness of intercultural communication among students studying abroad.

### Mediating Role of the Intergroup Anxiety

It is common knowledge that individuals often experience intergroup anxiety before interacting with people from a different culture (Stephan, 2014). In turn it amplifies cognitive and motivational biases and negative emotional reactions. Additionally, it lowers the evaluation of outgroup members; therefore high levels of intergroup anxiety is associated with low levels of contact with outgroup members (Stephan and Stephan, 1985). Many studies have indicated that intergroup anxiety is correlated with negative outgroup attitudes and negative emotions such as fear and anger (Stephan, 2014). Thus, it is not surprising that intergroup anxiety is associated with low quality of intercultural relationships (Greenland and Brown, 1999).

Additionally, Gudykunst’s AUM theory (1995) posits the impact of anxiety on intercultural interactions. It assumes that managing uncertainty and anxiety are central processes influencing the effectiveness of our communication with others (Gudykunst, 1995). Hence individuals can communicate effectively to the extent that they are able to manage their anxiety and accurately predict and explain others’ attitudes, feelings, and behaviors. Anxiety and uncertainty management, therefore, are the “basic” causes of effective communication. AUM theory assumes that the effects of other variables on communicative effectiveness are mediated through anxiety and uncertainty (Gudykunst, 1995).

Since intergroup anxiety predicts the effectiveness of intercultural communication; interpersonal mindfulness may therefore be related to the level of intergroup anxiety. It is well documented that the mindfulness-based interventions are effective for treating social anxiety (Goldin et al., 2017). Price-Blackshear et al. (2017) have found that mindfulness may help people regulate feelings of intergroup anxiety.

On the contrary, Harrison and Peacock (2009) have noted that increased intercultural contact can actually increase anxiety or decrease confidence in the short-term while new understanding and identities are constructed, particularly where mindfulness is stimulated. Students may worry about “mindful” forms of interactions in intercultural environments (Harrison and Peacock, 2009), further stimulating their levels of the intergroup anxiety. This contradiction needs to be clarified through empirical research.

Thus, *this study aims* to identify the interrelationship between interpersonal mindfulness, intergroup anxiety, and intercultural communication effectiveness among international students studying in Russian Universities.

Based on the positive role of the interpersonal mindfulness in the communication in previous research, the following hypotheses were proposed in this study.

*Hypothesis 1:* Interpersonal mindfulness has a positive relationship with intercultural communication effectiveness and a negative relationship with intergroup anxiety.

*Hypothesis 2:* Intergroup anxiety mediates the positive relationship between interpersonal mindfulness and intercultural communication effectiveness.

## Methods

### Sample

Participants were 337 (*M*_age_ = 22.93, *SD* = 3.11) international students (41.5% of females) from Russian universities. Based on the primary nationality the participants identified with, the demographic breakdown was as follows: Azerbaijan (1.7%), Ecuador (2.6%), China (13.6%), Mongolia (5.0%), Tajikistan (2.3%), Turkmenistan (39.0%), Uzbekistan (3.5%), and others – Afghanistan, Sudan, Syria, Vietnam, etc. (32.3%). Most respondents stay in Russia for 2–5 years (45.0%) or 1–2 years (36.2%), only 11.2% of them live in Russia for more than 5 years, and 7.6% – for less than 1 year. All of them were studying in the Russian language. Data were collected in Russian via an online survey service AnketologBox.

### Measurements

*Interpersonal mindfulness* was measured by *The Interpersonal Mindfulness Scale* (IMS; Pratscher et al., 2019). It assessed four aspects of mindfulness: attention to the present moment, awareness of self and others, non-judgmental acceptance, and non-reactivity in interpersonal interactions. The validation study that used Rasch analysis to investigate psychometric properties of the IMS was conducted by O.N. Medvedev et al. (2020) and found that the scale demonstrated robust reliability and internal validity. Research controlling for mindfulness as a trait, found that interpersonal mindfulness is associated with the interpersonal outcome of friendship quality (Pratscher et al., 2018). This Scale includes 27 items rated on a 5-point Likert scale (from 1 = almost always to 5 = almost never). Examples of the items are: “When I am with other people, I am aware of my moods and emotions” and “In tense moments with another person, I am aware of my feelings but do not get taken over by them.” Validity and reliability of the Russian version of *The Interpersonal Mindfulness* scale were tested by the Confirmatory factor Analysis. The one-factor model yielded good fit indices: χ^2^/df = 1.543; RMSEA = 0.040; TLI = 0.934; CFI = 0.995; SRMR = 0.044 (Hu and Bentler, 1999).

*Intergroup anxiety* was measured using ten items adapted from Stephan and Stephan (1985) and used Gudykunst and Nishida (2001). The items took the general form: “I felt ____ during my interaction with Russians”. The adjectives used were anxious, irritated, worried, etc. The items are rated on a 5-point Likert scale (from 1 = strongly disagree to 5 = strongly agree).

*Intercultural communication effectiveness* was measured using the eight items adapted from Gudykunst’s Perceived Effectiveness of Communication measure (Gudykunst and Nishida, 2001) and the General Self-Efficacy Scale (Scholz et al., 2002). Examples of the items are: “I felt competent when I communicated with Russians” and “I can solve most problems in communication with Russians.” The items are rated on a 5-point Likert scale (from 1 = disagree to 5 = strongly agree).

Russian versions of *The Intergroup Anxiety* and *The Intercultural Communication Effectiveness* scales have been tested in previous studies in Russia and found to have adequate validity and reliability (Gritsenko et al., 2021; Khukhlaev and Bratkina, 2021).

### Data Analysis

Descriptive analysis and correlations were conducted using the Statistical Package for Social Sciences (SPSS) 26.0., and mediation analyses were conducted using the PROCESS v. 3.3 macro for SPSS. We only included those respondents with no missing data on any of the variables. Following data imputation, we screened for univariate outliers based on the standard deviation (Tabachnick and Fidell, 2013) and multivariate outliers based on the MCD (Leys et al., 2018), and there were no outliers. No problems concerning the normality of the residuals, linearity, and homogeneity of variance between predictors and dependent variables were detected. All data were standardized following the recommendations of Hayes (2018).

We also added control variables such as gender, age, and length of stay in Russia because all of them potentially influence the research outcomes. Gender differences could influence the acculturation process (Güngör and Bornstein, 2009, 2013), intercultural competence (Solhaug and Kristensen, 2020), and intercultural adjustments of the international students (Tompkins et al., 2017). Likewise, age plays a great role in immigrants outcomes (Yeh, 2003; Morawa et al., 2020; Ma and Xia, 2021), and length of stay abroad is an important factor affecting international students’ communication skills (de Araujo, 2011; Jenny, 2013; Thomas, 2018).

To test the hypothesis from the PROCESS macro v3.4 (Hayes, 2018) model 4 was utilized (X = predictor; Y = outcome; M = mediators) with Õ as interpersonal mindfulness, Y as intercultural communication effectiveness, and M as intergroup anxiety.

## Results

Descriptive statistics, including the means, standard deviations, and zero-order correlations are presented in Table 1. Inspection of the means shows that, on average, participants reported an above-average level of intergroup anxiety and high level of interpersonal mindfulness and intercultural communication effectiveness. As expected, interpersonal mindfulness was positively associated with intercultural communication effectiveness and negatively with intergroup anxiety.

**TABLE 1:** Descriptive statistics and correlations between scales.

	Mean	SD	α	Interpersonal mindfulness	Intergroup anxiety
(1) Interpersonal mindfulness	3.43	0.54	0.83		
(2) Intergroup anxiety	2.37	0.74	0.74	−0.48*	
(3) Intercultural communication effectiveness	3.64	0.74	0.82	0.42*	−0.63*

N = 337; *p < 0.000.

To examine the plausibility of the hypotheses, based on recommendations from Hayes (2018), regression-based mediation analyses were conducted. Analyses on the full sample first revealed a significant total effect between the predictor (interpersonal mindfulness) and the outcome (intercultural communication effectiveness); *B* = 0.60, SE = 0.07, *p* < 0.000. Results then revealed that interpersonal mindfulness predicted intergroup anxiety; *B* = −0.64, SE = 0.07, *p* < 0.000; and that intergroup anxiety subsequently predicted intercultural communication effectiveness; *B* = –0.56, SE = 0.05, *p* ≤ 0.000.

To examine the significance of the indirect effect, we calculated bias corrected 95% confidence intervals (CI) of the indirect effects using 10,000 bootstrapped resamples. As “0” was not contained within the confidence intervals, the indirect effect of interpersonal mindfulness was significant; 95% CI (0.26, 0.47). The direct effect of interpersonal mindfulness on intercultural communication effectiveness was also significant; *B* = 0.24, SE = 0.07, *p* ≤ 0.000.

Moreover, though not the focus of the research hypotheses, there were three control variables in the mediation model: age, gender, and length of stay in Russia (Table 2). Only age was the negatively significant predictor of intergroup anxiety (*B* = −0.02, SE = 0.01, *p* ≤ 0.05).

**TABLE 2:** Path coefficients for relation proposed in hypotheses.

	Outcome: Intergroup anxiety					Outcome: Intercultural communication effectiveness				
	*B*	SE	*t*	Lower 95% CI	Upper 95% CI	*B*	SE	*t*	Lower 95% CI	Upper 95% CI
Control variables										
Age	−0.02*	0.01	−2.06	−0.05	−0.00	−0.01	0.01	−0.97	−0.03	0.01
Gender	−0.06	0.07	−0.82	−0.20	0.08	0.10	,06	1.51	−0.03	0.22
Length of stay in Russia	0.02	0.03	0.63	−0.04	0.08	0.00	0.02	0.35	−0.04	0.06
Predictor: Interpersonal Mindfulness	−0.64**	0.07	−9.66	−0.77	−0.51	0.24**	0.07	3.54	0.11	0.37
Mediator: Intergroup Anxiety	–	–	–	–	–	−0.56**	0.05	−0.11.56	−0.66	−0.46
R^2^	0.24					0.42				

N = 337. *p < 0.05; ** p < 0.001.

## Discussion

The current study examined the interrelationship between interpersonal mindfulness, intergroup anxiety, and intercultural communication effectiveness. Consistent with the first hypothesis, interpersonal mindfulness was significantly, negatively associated with intergroup anxiety. Also, the second hypothesis was supported, such that intergroup anxiety mediates the effect of interpersonal mindfulness on intercultural communication effectiveness.

A possible reason for this result may be that international students who pay more attention to the present moment while communicating with Russians, have lower anticipation of the negative results of the communication; therefore, they feel less anxiety in intercultural interactions. Research has documented that mindfulness may interrupt the cycle of avoidance and de-motivation based on the anticipatory fear of negative evaluation apprehension (Carlton et al., 2020).

Another aspect of the interpersonal mindfulness is awareness of self and others during interpersonal interactions (Pratscher et al., 2019). In intercultural communication it means that international students with high level of the interpersonal mindfulness demonstrate higher levels of the intercultural sensitivity. According to one of the most popular intercultural competence model, the Developmental Model of Intercultural Sensitivity (Bennett, 2017), intercultural sensitivity is “the ability to discriminate and experience relevant cultural differences.” Hammer et al. (2003, p. 422) explain it as “the ability to think and act in interculturally appropriate ways.” Previous research marked that intercultural sensitivity is negatively correlated with intergroup threat (Lupano Peruginni and Castro Solano, 2015). International students who are mindful, are more attentive and have better observational skills, particularly observing things appropriate for adaptation in a new culture. It decreases the uncertainty of the intercultural communication which in turn decreases the level of anxiety (Gudykunst and Nishida, 2001).

Other aspects of interpersonal mindfulness are non-reactivity and non-judgmental acceptance during conversations (Pratscher et al., 2019). These components are interrelated and refer to both meta-cognition awareness and affective attunement facets of mindfulness, proposed by Ting-Toomey and Dorjee (2015). Mindful people can monitor and modify reactive cognitive schemata to understand the new cultural environment through awareness of the context, plan the implications of their own behavior, and adjust habituated mental patterns to new mental maps and novel cultural scripts (Thomas, 2006). In this approach the concept of mindfulness is understood as the metacognitive strategy that links meta-thinking, knowledge, and behavioral flexibility, and is considered as a key component of Cultural Intelligence (CQ). Moreover, CQ is negatively correlated with intergroup anxiety (Presbitero and Attar, 2018) and CQ level among managers in multinational companies was negatively associated with the level of anxiety experienced in cross-cultural interactions (Bücker et al., 2014).

People can use mindful attunement process to create a more differentiated and complex view of the dissimilar, stereotyped group (Ting-Toomey and Dorjee, 2015). In a previous research, acceptance was negatively associated with state interracial intergroup anxiety (Oyler et al., 2021).

In sum, all factors of interpersonal mindfulness have well-described and evidence-based mechanisms for decreasing anxiety in intercultural communication. Therefore, mediation mechanism of the impact of interpersonal mindfulness on intercultural communication effectiveness through intergroup anxiety is acknowledged for international students.

In addition to indirect effect, we also found a direct effect of interpersonal mindfulness on intercultural communication effectiveness; indicating other causal mechanisms of this relation. Plausible causal paths of this effect may lead to different aspects explained in the mindfulness-intergroup bias research such as stress-reduction (Kang et al., 2014), promoting empathy (Hunsinger et al., 2014), and cognitive flexibility (Lillis and Hayes, 2007).

### Conclusion, Limitations, and Future Directions

In summary, the present study offers evidence in support of interpersonal mindfulness as antecedent to intercultural communication effectiveness among international students.

This study extends the literature on interpersonal mindfulness research using data obtained on a sample of international students studying at Russian universities. The results of this study suggests that the inclusion of both interpersonal mindfulness-based interventions and intergroup anxiety reduction trainings, in psychological support programs for international students will lead to increased effectiveness in intercultural communication. This, in turn, can lead to better intercultural adaptation of students from different countries in the multicultural educational space of international universities.

Despite presenting significant results, this study has some limitations. First, the study examined and measured only interpersonal mindfulness and did not control for the level of mindfulness as a general trait. Second, the sample is not balanced in terms of composition by nationality of international students; it includes an unequal number of students from different countries, including from the republics of the former Soviet Union. Additionally, students in this study had different levels of Russian language proficiency; this can affect their effectiveness in intercultural communication. Finally, the use of a self-reported technique for analysis of the intercultural communication effectiveness may have led to subjectivity in responses.

Summing up all the findings and limitations of this research, we have determined the following future directions: (1) expansion of the sample and dividing it into subsamples of international students from different countries; (2) using additional techniques for assessment of variables such as expert assessment for intercultural communication effectiveness and adapted scales in native languages of students; (3) development and addition of programs for psychological and pedagogical support toward intercultural adjustment process for international students in Russian universities based on the research findings.

## Data Availability Statement

The raw data supporting the conclusions of this article will be made available by the authors, without undue reservation.

## Ethics Statement

The studies involving human participants were reviewed and approved by Ethics Committee of RUDN University (Peoples’ Friendship University of Russia). The patients/participants provided their written informed consent to participate in this study.

## Author Contributions

OK: data analysis and text writing. IN: data collection and text writing. AC: data collection. All authors contributed to the article and approved the submitted version.

## Conflict of Interest

The authors declare that the research was conducted in the absence of any commercial or financial relationships that could be construed as a potential conflict of interest.

## Publisher’s Note

All claims expressed in this article are solely those of the authors and do not necessarily represent those of their affiliated organizations, or those of the publisher, the editors and the reviewers. Any product that may be evaluated in this article, or claim that may be made by its manufacturer, is not guaranteed or endorsed by the publisher.

## Abbreviations

AUM: anxiety/uncertainty management
CQ: cultural intelligence
IMS: The Interpersonal Mindfulness Scale
SPSS: Statistical Package for Social Sciences.

## References

[B1] Abramova M. O. Filkina A. V. Sukhushina E. V. (2021). Challenges to internationalization in Russian higher education: the impact of the COVID-19 pandemic on the international student experience. *Vopr. Obrazovaniya Educ. Stud. Mosc.* 4 117–146. 10.17323/1814-9545-2021-4-117-146

[B2] Aresi G. Moore S. C. Marta E. (2021). The health behaviours of European study abroad students sampled from forty-two countries: data from a three-wave longitudinal study. *Data Br.* 38:107285. 10.1016/j.dib.2021.107285 34458517 PMC8377420

[B3] Bennett M. J. (2017). “Developmental model of intercultural sensitivity,” in *The international Encyclopedia of Intercultural Communication*, ed. Kim Y. Y. (Hoboken, N J: John Wiley & Sons, Inc). 10.1002/9781118783665.ieicc0182

[B4] Bierwiaczonek K. Waldzus S. (2016). Socio-cultural factors as antecedents of cross-cultural adaptation in expatriates, international students, and migrants: a review. *J. Cross Cult. Psychol.* 47 767–817. 10.1177/0022022116644526

[B5] Bücker J. J. L. E. Furrer O. Poutsma E. Buyens D. (2014). The impact of cultural intelligence on communication effectiveness, job satisfaction and anxiety for Chinese host country managers working for foreign multinationals. *Int. J. Hum. Resour. Manag.* 25 2068–2087. 10.1080/09585192.2013.870293

[B6] Bui H. T. N. Selvarajah C. Vinen D. G. Meyer D. (2021). Acculturation: role of student–University alignment for international student psychological adjustment. *J. Stud. Int. Educ.* 25 546–565. 10.1177/1028315320964286

[B7] Burgoon J. K. Berger C. R. Waldron V. R. (2000). Mindfulness and interpersonal communication. *J. Soc. Issues* 56 105–127. 10.1111/0022-4537.00154

[B8] Cao C. Meng Q. (2022). A systematic review of predictors of international students’ cross-cultural adjustment in china: current knowledge and agenda for future research. *Asia Pac. Educ. Rev.* 23 45–67. 10.1007/s12564-021-09700-1

[B9] Carlton C. N. Sullivan-Toole H. Strege M. V. Ollendick T. H. Richey J. A. (2020). Mindfulness-based interventions for adolescent social anxiety: a unique convergence of factors. *Front. Psychol.* 11:1783. 10.3389/fpsyg.2020.01783 32774320 PMC7387717

[B10] Chebotareva E. J. (2011). Intercultural adaptation to Russia of students from Asia, Africa, Latin America and the middle East. *RUDN J. Psychol. Pedagog.* 3 6–11.

[B11] de Araujo A. A. (2011). Adjustment issues of international students enrolled in American colleges and universities: a review of the literature. *High. Educ. Stud.* 1 2–8. 10.5539/hes.v1n1p2

[B12] Don B. P. (2020). Mindfulness predicts growth belief and positive outcomes in social relationships. *Self Identity* 19 272–292. 10.1080/15298868.2019.1571526

[B13] Goldin P. R. Morrison A. S. Jazaieri H. Heimberg R. G. Gross J. J. (2017). Trajectories of social anxiety, cognitive reappraisal, and mindfulness during an RCT of CBGT versus MBSR for social anxiety disorder. *Behav Res. Ther.* 97, 1–13. 10.1016/j.brat.2017.06.001 28654771 PMC5600696

[B14] Greenland K. Brown R. (1999). Categorization and intergroup anxiety in contact between British and Japanese nationals. *Eur. J. Soc. Psychol.* 29 503–521. 10.1002/(SICI)1099-0992(199906)29:4<503::AID-EJSP941<3.0.CO;2-Y

[B15] Gritsenko V. V. Khukhlaev OE Zinurova R. I Konstantinov V. V. Kulesh ÅV. Malyshev I. V. (2021). Intercultural competence as a predictor of adaptation of foreign students. *Cultur. Hist. Psychol.* 17 103–112. 10.17759/chp.2021170114

[B16] Gudykunst W. B. (1995). “Anxiety/uncertainty management (AUM) theory: current status,” in *Intercultural Communication Theory*, ed. Wiseman R. L. (Thousand Oaks, CA: Sage), 8–57.

[B17] Gudykunst W. B. (1998). Applying anxiety\uncertainty management (AUM) theory to intercultural adjustment training. *Int. J. Intercult. Relat.* 22 227–250. 10.1016/S0147-1767(98)00005-4

[B18] Gudykunst W. B. Nishida T. (2001). Anxiety, uncertainty, and perceived effectiveness of communication across relationships and cultures. *Int. J. Intercult. Relat.* 25 55–71. 10.1016/S0147-1767(00)00042-0

[B19] Güngör D. Bornstein M. H. (2009). Gender, development, values, adaptation, and discrimination in acculturating adolescents: the case of turk heritage youth born and living in Belgium. *Sex Roles* 60 537–548. 10.1007/s11199-008-9531-2

[B20] Güngör D. Bornstein M. H. (2013). “Gender and developmental pathways of acculturation and adaptation in immigrant adolescents,” in *Gender Roles in Immigrant Families*, eds Chuang S. S. Tamis-LeMonda C. S. (New York, NY: Springer), 177–190. 10.1007/978-1-4614-6735-9_11

[B21] Hammer M. R. Bennett M. J. Wiseman R. (2003). Measuring intercultural sensitivity: the intercultural development inventory. *Int. J. Intercult. Relat.* 27 421–443. 10.1016/S0147-1767(03)00032-4

[B22] Harrison N. Peacock N. (2009). Cultural distance, mindfulness and passive xenophobia: using integrated threat theory to explore home higher education students’ perspectives on “internationalisation at home.”. *Br. Educ. Res. J.* 36 877–902. 10.1080/01411920903191047

[B23] Hayes A. F. (2018). *Introduction to Mediation, Moderation, and Conditional Process Analysis.* New York, NY: The Guilford Press.

[B24] Hu L. Bentler P. M. (1999). Cutoff criteria for fit indices in covariance structure analysis: conventional criteria versus new alternatives. *Struct. Equ. Modeling* 6 1–55. 10.1080/10705519909540118

[B25] Hunsinger M. Livingston R. Isbell L. (2014). Spirituality and intergroup harmony: meditation and racial prejudice. *Mindfulness* 5 139–144. 10.1007/s12671-012-0159-5

[B26] Jankowski P. J. Sandage S. J. (2014). Meditative prayer and intercultural competence: empirical test of a differentiation-based model. *Mindfulness* 5 360–372. 10.1007/s12671-012-0189-z

[B27] Jenny M. (2013). International student migration: outcomes and implications. *J. Int. Stud.* 3 167–181. 10.32674/jis.v3i2.509

[B28] Kang Y. Gray J. R. Dovidio J. F. (2014). The nondiscriminating heart: lovingkindness meditation training decreases implicit intergroup bias. *J. Exp. Psychol. Gen.* 143 1306–1313. 10.1037/a0034150 23957283

[B29] Kanjera S. (2021). The impact of COVID-19 on the internationalization of higher education. *Int. Res. Higher Educ.* 6:25. 10.5430/irhe.v6n1p25

[B30] Karremans J. C. Schellekens M. P. J. Kappen G. (2017). Bridging the sciences of mindfulness and romantic relationships. *Pers. Soc. Psychol. Rev.* 21 29–49. 10.1177/1088868315615450 26563236

[B31] Kashima E. S. Greiner T. Sadewo G. Ampuni S. Helou L. Nguyen V. A. (2017). Open- and closed-mindedness in cross-cultural adaptation: the roles of mindfulness and need for cognitive closure. *Int. J. Intercult. Relat.* 59 31–42. 10.1016/j.ijintrel.2017.05.001

[B32] Khukhlaev O. E. Bratkina M. (2021). Anxiety and uncertainty in intercultural communication: experimental research. *Russ. Psych. J.* 18 78–90. 10.21702/rpj.2021.4.6

[B33] Khukhlaev O. E. Gritsenko V. V. Pavlova O. S. Tkachenko N. V. Usubyan S. A. Shorokhova V. A. (2020). Comprehensive model of intercultural competence: theoretical substantiation. *RUDN J. Psychol. Pedagog.* 17 9–25. 10.22363/2313-1683-2020-17-1-13-28

[B34] Leys C. Klein O. Dominicy Y. Ley C. (2018). Detecting multivariate outliers: use a robust variant of the Mahalanobis distance. *J. Exp. Soc. Psychol.* 74 150–156. 10.1016/j.jesp.2017.09.011

[B35] Lillis J. Hayes S. C. (2007). Applying acceptance, mindfulness, and values to the reduction of prejudice: a pilot study. *Behav. Modif.* 31 389–411. 10.1177/0145445506298413 17548537

[B36] Lupano Peruginni M. L. Castro Solano A. (2015). Intergroup anxiety, cultural sensitivity and socio-cultural diverse leaders’ effectiveness Ansiedad intergrupal, sensibilidad cultural y research. *Int. J. Psychol. Res.* 8 36–45. 10.21500/20112084.643

[B37] Ma Z. Xia Y. (2021). Acculturation strategies, age at migration, and self-rated health: an empirical study on internal migrants in China. *Soc. Sci. Res.* 93:102487. 10.1016/j.ssresearch.2020.102487 33308690

[B38] Mao Y. Ji H. Wang R. (2022). Expectation and reality: international students’ motivations and motivational adjustments to sustain academic journey in Chinese universities. *Front. Psychol.* 13:833407. 10.3389/fpsyg.2022.833407 35282185 PMC8912981

[B39] Matera C. Catania M. A. (2021). Correlates of international students’ intergroup intentions and adjustment: the role of metastereotypes and intercultural communication apprehension. *Int. J. Intercult. Relat.* 82 288–297. 10.1016/j.ijintrel.2021.04.011

[B40] Medvedev O. N. Pratscher S. D. Bettencourt A. (2020). Psychometric evaluation of the interpersonal mindfulness scale using Rasch analysis. *Mindfulness* 11 2007–2015. 10.1007/s12671-020-01415-5

[B41] Morawa E. Brand T. Dragano N. Jöckel K. H. Moebus S. Erim Y. (2020). Associations between acculturation, depressive symptoms, and life satisfaction among migrants of turkish origin in germany: gender- and generation-related aspects. *Front. Psychiatr.* 11:1–10. 10.3389/fpsyt.2020.00715 32848908 PMC7406783

[B42] Nadeem M. U. Koschmann M. A. (2021). Does mindfulness moderate the relationship between anxiety, uncertainty, and intercultural communication effectiveness of the students in Pakistan? *Curr. Psychol.* 1–13. 10.1007/s12144-021-01429-9

[B43] Neuliep J. W. (2012). The relationship among intercultural communication apprehension, ethnocentrism, uncertainty reduction, and communication satisfaction during initial intercultural interaction: an extension of anxiety and uncertainty management (AUM) theory. *J. Intercult. Commun. Res.* 41 1–16. 10.1080/17475759.2011.623239

[B44] Novikova I. A. Novikov A. L. (2015). Relation between communicative tolerance and intercultural adaptation in international students. *Mediterr. J. Soc. Sci.* 6 109–116. 10.5901/mjss.2015.v6n2s2p109

[B45] Oyler D. L. Price-Blackshear M. A. Pratscher S. D. Bettencourt B. A. (2021). Mindfulness and intergroup bias: a systematic review. *Group Process. Intergroup Relat.* 136843022097869. 10.1177/1368430220978694

[B46] Pekerti A. A. van de Vijver F. J. R. Moeller M. Okimoto T. G. Edwards M. R. (2021). A peer mentoring social learning perspective of cross-cultural adjustment: the rapid-acculturation mateship program. *Int. J. Intercult. Relat.* 84 276–299. 10.1016/j.ijintrel.2021.08.010

[B47] Pratscher S. D. Rose A. J. Markovitz L. Bettencourt A. (2018). Interpersonal mindfulness: investigating mindfulness in interpersonal interactions, co-rumination, and friendship quality. *Mindfulness* 9 1206–1215. 10.1007/s12671-017-0859-y

[B48] Pratscher S. D. Wood P. K. King L. A. Bettencourt B. A. (2019). Interpersonal mindfulness: scale development and initial construct validation. *Mindfulness* 10 1044–1061. 10.1007/s12671-018-1057-2

[B49] Presbitero A. Attar H. (2018). Intercultural communication effectiveness, cultural intelligence and knowledge sharing: extending anxiety-uncertainty management theory. *Int. J. Intercult. Relat.* 67 35–43. 10.1016/j.ijintrel.2018.08.004

[B50] Price-Blackshear M. A. Kamble S. V. Mudhol V. Sheldon K. M. Ann Bettencourt B. (2017). Mindfulness practices moderate the association between intergroup anxiety and outgroup attitudes. *Mindfulness* 8, 1172–1183. 10.1007/s12671-017-0689-y

[B51] Scholz U. Gutiérrez Doña B. G. Sud S. Schwarzer R. (2002). Is general self-efficacy a universal construct? *Eur. J. Psychol. Assess.* 18 242–251. 10.1027//1015-5759.18.3.242

[B52] Solhaug T. Kristensen N. N. (2020). Gender and intercultural competence: analysis of intercultural competence among upper secondary school students in Denmark and Norway. *Educ. Psychol.* 40 120–140. 10.1080/01443410.2019.1646410

[B53] Stephan W. G. (2014). Intergroup anxiety: theory, research, and practice. *Pers. Soc. Psychol. Rev.* 18 239–255. 10.1177/1088868314530518 24815215

[B54] Stephan W. G. Stephan C. W. (1985). Intergroup anxiety. *J. Soc. Issues* 41 157–175. 10.1111/j.1540-4560.1985.tb01134.x

[B55] Tabachnick B. G. Fidell L. S. (2013). *Using Multivariate Statistics*, 6th Edn. Boston, MA: Pearson.

[B56] Thomas D. C. (2006). Domain and development of cultural intelligence: the importance of mindfulness. *Group Organ. Manag.* 31 78–99. 10.1177/1059601105275266

[B57] Thomas F. (2018). Length of stay and acculturative stress of international students: empirical study. *Int. J. Pure Appl. Math.* 120 10253–10264.

[B58] Ting-Toomey S. Dorjee T. (2015). “Intercultural and intergroup communication competence: toward an integrative perspective,” in *Communication Competence*, eds Hannawa A. F. Spitzberg B. H. (Berlin: De Gruyter Mouton), 503–508. 10.1515/9783110317459-021

[B59] Tompkins A. Cook T. Miller E. LePeau L. A. (2017). Gender influences on students’ study abroad participation and intercultural competence. *J. Stud. Aff. Res. Pract.* 54 204–216. 10.1080/19496591.2017.1284671

[B60] Xiong Y. Prasath P. R. Zhang Q. Jeon L. (2022). A mindfulness-based well-being group for international students in higher education: a pilot study. *J. Counsel. Dev.* 10.1002/jcad.12432

[B61] Yeh C. J. (2003). Age, acculturation, cultural adjustment, and mental health symptoms of Chinese, Korean, and Japanese immigrant youths. *Cultur. Divers. Ethnic Minor. Psychol.* 9 34–48. 10.1037/1099-9809.9.1.34 12647324

